# Supplementary material for: HIVprotI: an integrated web based platform for prediction and design of HIV proteins inhibitors
Source: J Cheminform. 2018 Mar 9;10:12. doi: 10.1186/s13321-018-0266-y (PMC5845081; doi:10.1186/s13321-018-0266-y)
Supplement: Supplementary file 2 — Additional file 2. Source code of HIVProtI web server. [file 13321_2018_266_MOESM2_ESM.zip › HIVprotI_Source-code/hivprotI/weka-3-6-6/doc/weka/core/AttributeLocator.html]

AttributeLocator


---


|  |  |  |
| --- | --- | --- |
| **Overview** \| **Package** \| **Class** \| **Tree** \| **Deprecated** \| **Index** \| **Help** \| **Weka's home** | |  |
| **PREV CLASS**   **NEXT CLASS** | **FRAMES**    **NO FRAMES**     **All Classes** |  |
| SUMMARY: NESTED \| FIELD \| CONSTR \| METHOD | DETAIL: FIELD \| CONSTR \| METHOD |  |


---


## weka.core Class AttributeLocator

```
java.lang.Object
  weka.core.AttributeLocator
```

**All Implemented Interfaces:**: java.io.Serializable, java.lang.Comparable<AttributeLocator>, RevisionHandler

**Direct Known Subclasses:**: RelationalLocator, StringLocator

---

``` public class AttributeLocator extends java.lang.Object implements java.io.Serializable, java.lang.Comparable<AttributeLocator>, RevisionHandler ```

This class locates and records the indices of a certain type of attributes,
recursively in case of Relational attributes.

**Version:**
:   $Revision: 1.4 $

**Author:**
:   fracpete (fracpete at waikato dot ac dot nz)

**See Also:**: `Attribute.RELATIONAL`, Serialized Form

---

**Constructor Summary**

`AttributeLocator(Instances data,
int type)`
  
          Initializes the AttributeLocator with the given data for the specified
type of attribute.

`AttributeLocator(Instances data,
int type,
int[] indices)`
  
          initializes the AttributeLocator with the given data for the specified
type of attribute.

`AttributeLocator(Instances data,
int type,
int fromIndex,
int toIndex)`
  
          Initializes the AttributeLocator with the given data for the specified
type of attribute.


| **Method Summary** | |
| --- | --- |
| `int` | `compareTo(AttributeLocator o)`             Compares this object with the specified object for order. |
| `boolean` | `equals(java.lang.Object o)`             Indicates whether some other object is "equal to" this one. |
| `int` | `getActualIndex(int index)`             returns actual index in the Instances object. |
| `int[]` | `getAllowedIndices()`             returns the indices that are allowed to check for the attribute type |
| `int[]` | `getAttributeIndices()`             Returns the indices of the attributes. |
| `Instances` | `getData()`             returns the underlying data |
| `AttributeLocator` | `getLocator(int index)`             Returns the AttributeLocator at the given index. |
| `int[]` | `getLocatorIndices()`             Returns the indices of the AttributeLocator objects. |
| `java.lang.String` | `getRevision()`             Returns the revision string. |
| `int` | `getType()`             returns the type of attribute that is located |
| `java.lang.String` | `toString()`             returns a string representation of this object |

**Methods inherited from class java.lang.Object**

`getClass, hashCode, notify, notifyAll, wait, wait, wait`

**Constructor Detail**

### AttributeLocator

```
public AttributeLocator(Instances data,
                        int type)
```

:   Initializes the AttributeLocator with the given data for the specified
    type of attribute. Checks all attributes.

    **Parameters:**: `data` - the data to work on: `type` - the type of attribute to locate

---


### AttributeLocator

```
public AttributeLocator(Instances data,
                        int type,
                        int fromIndex,
                        int toIndex)
```

:   Initializes the AttributeLocator with the given data for the specified
    type of attribute. Checks only the given range.

    **Parameters:**: `data` - the data to work on: `type` - the type of attribute to locate: `fromIndex` - the first index to inspect (including): `toIndex` - the last index to check (including)

---


### AttributeLocator

```
public AttributeLocator(Instances data,
                        int type,
                        int[] indices)
```

:   initializes the AttributeLocator with the given data for the specified
    type of attribute. Checks only the given attribute indices.

    **Parameters:**: `data` - the data to work on: `type` - the type of attribute to locate: `indices` - the attribute indices to check


**Method Detail**

### getType

```
public int getType()
```

:   returns the type of attribute that is located

    :   **Returns:**: the type of attribute

---


### getAllowedIndices

```
public int[] getAllowedIndices()
```

:   returns the indices that are allowed to check for the attribute type

    :   **Returns:**: the indices that are checked for the attribute type

---


### getData

```
public Instances getData()
```

:   returns the underlying data

    :   **Returns:**: the underlying Instances object

---


### getActualIndex

```
public int getActualIndex(int index)
```

:   returns actual index in the Instances object.

    :   **Parameters:**: `index` - the index in the m\_AllowedIndices array **Returns:**: the actual index in the instances object

---


### getAttributeIndices

```
public int[] getAttributeIndices()
```

:   Returns the indices of the attributes. These indices are referring
    to the m\_AllowedIndices array, not the actual indices in the Instances
    object.

    :   **Returns:**: the indices of the attributes **See Also:**: `getActualIndex(int)`

---


### getLocatorIndices

```
public int[] getLocatorIndices()
```

:   Returns the indices of the AttributeLocator objects. These indices are
    referring to the m\_AllowedIndices array, not the actual indices in the
    Instances object.

    :   **Returns:**: the indices of the AttributeLocator objects **See Also:**: `getActualIndex(int)`

---


### getLocator

```
public AttributeLocator getLocator(int index)
```

:   Returns the AttributeLocator at the given index. This index refers to
    the index of the m\_AllowedIndices array, not the actual Instances object.

    :   **Parameters:**: `index` - the index of the locator to retrieve **Returns:**: the AttributeLocator at the given index

---


### compareTo

```
public int compareTo(AttributeLocator o)
```

:   Compares this object with the specified object for order. Returns a
    negative integer, zero, or a positive integer as this object is less
    than, equal to, or greater than the specified object. Only type and
    indices are checked.

    :   **Specified by:**: `compareTo` in interface `java.lang.Comparable<AttributeLocator>`
    :   **Parameters:**: `o` - the object to compare with **Returns:**: -1 if less than, 0 if equal, +1 if greater than the given object

---


### equals

```
public boolean equals(java.lang.Object o)
```

:   Indicates whether some other object is "equal to" this one. Only type
    and indices are checked.

    :   **Overrides:**: `equals` in class `java.lang.Object`
    :   **Parameters:**: `o` - the AttributeLocator to check for equality **Returns:**: true if the AttributeLocators have the same type and indices

---


### toString

```
public java.lang.String toString()
```

:   returns a string representation of this object

    :   **Overrides:**: `toString` in class `java.lang.Object`
    :   **Returns:**: a string representation

---


### getRevision

```
public java.lang.String getRevision()
```

:   Returns the revision string.

    :   **Specified by:**: `getRevision` in interface `RevisionHandler`
    :   **Returns:**: the revision


---


|  |  |  |
| --- | --- | --- |
| **Overview** \| **Package** \| **Class** \| **Tree** \| **Deprecated** \| **Index** \| **Help** \| **Weka's home** | |  |
| **PREV CLASS**   **NEXT CLASS** | **FRAMES**    **NO FRAMES**     **All Classes** |  |
| SUMMARY: NESTED \| FIELD \| CONSTR \| METHOD | DETAIL: FIELD \| CONSTR \| METHOD |  |


---
